# Mentoring upcoming researchers for non-communicable diseases’ research and practice in Malawi

**DOI:** 10.1186/s13063-020-05006-6

**Published:** 2021-01-19

**Authors:** Adamson S. Muula, Mina C. Hosseinipour, Martha Makwero, Johnstone Kumwenda, Prosper Lutala, Mary Mbeba, Sarah Rylance, Collins Mitambo, Moffat Nyirenda

**Affiliations:** 1 College of Medicine, University of Malawi, Blantyre, Malawi; 2 UNC Project, Lilongwe, Malawi; 3 Kamuzu College of Nursing, University of Malawi, Blantyre, Malawi; 4 Malawi-Liverpool-Wellcome Trust (MLW) Trust, Clinical Research Programme, University of Malawi, Blantyre, Malawi; 5 Malawi Ministry of Health and Population, Lilongwe, Malawi

**Keywords:** Capacity building, Malawi, Mentorship, Non-communicable diseases, Research supervision

## Abstract

The Malawi College of Medicine and its partners are building non-communicable diseases’ (NCDs’) research capacity through a grant from the National Heart, Lung and Blood Institute (NHLBI) of the National Institutes of Health. Several strategies are being implemented including research mentorship for junior researchers interested to build careers in NCDs’ research. In this article, we present the rationale for and our experiences with this mentorship program over its 2 years of implementation. Lessons learned and the challenges are also shared.

## Introduction and background

Africa is experiencing an increasing prevalence of non-communicable diseases (NCDs) [1]. Addressing the rising NCD burden in sub-Saharan Africa must be considered in regard to epidemiological context of each country. The large uncertainties around the estimates of the disease burden in sub-Saharan Africa, varying data availability between and within countries, make comparisons on burden and risk factors difficult to interpret and inform implementation of evidence-based interventions in the local context [2]. Consistent with findings from other settings, the prevalence and risk factors for hypertension and diabetes in Malawi is high with estimates ranging from 15.8 to 32.9% and from 2.4 to 5.6% respectively. However, out of the 130 studies reviewed in 2018 by the Lancet Non-Communicable Diseases and Injury (NCDI) poverty commission for Malawi, the majority of studies conducted in Malawi fell into three groups largely injuries, neoplasms, and cardiovascular diseases. Far fewer publications focused on digestive, neurological, and respiratory disorders, highlighting the lack of research into these conditions.

The lack of data and research in part emanates from the fact that the few available researchers are burdened with conducting research in communicable diseases, although capacity for NCD research is developing. Physician investigators may also be burdened with provision of clinical services within a context of severe human resources for health (HRH) shortages. In order to feed into the pipeline of producing highly trained and qualified researchers, there is need to build research capacity from graduate education, post-doctoral placement, and mentored research of junior faculty. In this paper, we focus on the mentored research component of the NCD-BRITE consortium, a Malawian consortium funded by the National Heart, Lung and Blood Institute.

## The NCD-BRITE initiative

In Malawi, several initiatives have begun addressing the NCD challenge, which have often utilized existing infectious disease infrastructure. It is crucial to carefully leverage these synergies to maximize their impact. The NCD-BRITE (Building Research Capacity, Implementation, and Translation Expertise for Non-Communicable Diseases), launched in February 2018, is a transdisciplinary consortium that brings together key research institutions, the Ministry of Health and Population, and other stakeholders to build long-term, sustainable, NCD-focused implementation research capacity. Led by University of Malawi-College of Medicine, University of North Carolina, and previously Dignitas International, NCD-BRITE’s specific aims are to conduct detailed assessments of the burden and risk factors of common NCDs [1]; assess the research infrastructure needed to inform, implement, and evaluate NCD interventions; create a national implementation research agenda for the priority NCDs; and develop NCD-focused implementation research capacity through short courses, mentored research awards, and an internship placement program. The capacity-building activities are purposely designed around the University of Malawi-College of Medicine, its affiliates, and Ministry of Health and Population to ensure sustainability. The overall goal, by the end of the grant award, is to create a national, actionable, implementation research agenda for NCD prioritized in this consortium, namely cardiovascular diseases, diabetes mellitus, asthma, and chronic obstructive pulmonary disease [3, 4].

## The rationale for mentored research

Mentorship is a proven strategy to scientific success for junior researchers [5]. Initially, graduate school provides the education and some training for students to design and implement a fully independent or semi-independent study. The latter may be embedded in a larger funded study in which a principal investigator permits the graduate student to obtain guided experience. In this setting, the principal investigator serves as an invested mentor as the goals of both individuals are closely aligned. In Malawi, due to scarcity of large funded studies, many graduate students conduct modest, self-funded studies as the basis of their thesis. In this scenario, the student solicits mentors with potential interest in the subject matter although not a primary focus. Though such studies allow the student to obtain skills in protocol development in readiness for ethics or IRB approvals, the individual may not gain the sufficient skills for subsequent grant funding proposal development. The challenges faced by young Malawian researchers seem to be similar to those from other African nations. These challenges include scarcity of mentors, lack of funding, lack of writing skills, lack of motivation, and low demand for research by policymakers.

The lack of support for early stage investigators in low- and middle-income countries interested in the global NCD field has resulted in inadequate funding opportunities for research, insufficient training in advanced research methodology and data analysis, lack of mentorship in manuscript and grant writing, and meager institutional support for developing, submitting, and administering research applications and awards. While such limited experiences may be the case for many graduate students across the world, the almost non-existence of postdoctoral placements for low-middle-income countries (LMIC) investigators means that many research careers do not blossom past the graduate education stage. The junior researcher mentored research component of our NCD-BRITE initiative provides the opportunity for individuals to obtain such research capacity building support. The cornerstone of the mentored research program includes identification of an invested mentorship team and adequate support for the proposed research.

## The NCD mentored research program

A primary focus of the NCD-BRITE program includes a strong understanding of implementation science, the mentored grants therefore requires an emphasis in this discipline. In year 1, based on a previous model used in the Fogarty Global Health Fellows program and the Fogarty Sponsored Malawi HIV implementation Scientist Training program, we solicited implementation science grant applications from interested individuals based at one of the NCD BRITE consortia partners in Malawi. This included national advertisement and local promotion of the call for applications among NCD BRITE Partner websites, notices on boards and email communication. Required application materials included a personal statement of career interests, a 2-page research proposal focused on implementation science of NCDs, 2 letters of support from potential mentors, and relevant bio-sketch. Grant applications were reviewed by 3 reviewers and scored according to criteria aligned to NIH career development criteria. Top grant application were funded.

Following the selection and with support from the identified mentors, the mentees are exposed to:

- Appropriate Short Course Programs including Research methods workshops, Statistics, Scientific Writing, and Introduction to Implementation Science. These would normally take 1 week long including practical and individual consultation sessions
- Protocol development
- Research grant budget preparation
- IRB submission
- Study implementation
- Data analysis
- Scientific writing including manuscripts and subsequent grants

## Competencies aimed for

Nine core global health research mentoring competencies have been identified [6]. These include maintaining effective communication; aligning expectations with reasonable goals and objectives; assessing and providing skills and knowledge for success; addressing diversity; fostering independence; promoting professional development; promoting professional integrity and ethical conduct; overcoming resource limitations; and fostering institutional change. We are working to ensure that throughout and at the end of the mentoring, we should be able to document and report on how we have paid attention to these goals. Therefore, both as a part of the grant selection process and the ongoing evaluation, we emphasize the importance of the mentor/mentee relationship. While a primary mentor is generally assigned to review these elements, we encourage a mentorship team as a single mentor may not possess all required expertise for a mentee’s development.

## Selected topics for research

The Malawi NCD-BRITE initiative has prioritized the following conditions or diseases and their complications: hypertension, heart disease, diabetes, asthma, and chronic obstructive pulmonary conditions. To date, 13 mentored research grants have been awarded and all these are still ongoing. The following research topics provide a sample of the selected topics for support from the NCD BRITE consortium.

- Brief behavior change counseling in lifestyle risk for diabetes-hypertension patients in Mangochi-southern Malawi
- Factors affecting optimal asthma management in adult patients accessing care
- Experiences and perceptions of delivery of asthma education sessions by non-medical personnel
- Assessing the clinical profile, risk aversion practices, and outcomes of diabetic foot ulceration
- Personal, behavioral, and environmental factors associated with self-management among diabetic patients attending the diabetic clinic at Queen Elizabeth Central Hospital (QECH)
- Physical activity and healthy diet, an intervention for hypertension prevention in Ndirande, Malawi—Quasi-experimental study
- Effectiveness of training mid-level eye care workers in diabetic retinopathy screening using a direct ophthalmoscope

## Lessons learnt

For the first 2 years of NCD-BRITE implementation and mentorship initiative, several lessons have been learned. Firstly, each time we have advertised for the mentored research opportunities, there have not been a large number of applicants. Yet we do advertise nationally and very few applications are received. There are several reasons for this. Communicable diseases have long been the focus of research in Malawi and funded research projects in which to embed trainee projects is much greater. Existing NCD research opportunities are much less and therefore interest may be lower. Additionally, the NCD BRITE program called for a focus on implementation science which is a new concept in the Malawian context and may have been a deterrent. Mentored research initiatives may be a new initiative that may become popular with time, and successful experiences are shared widely by those who graduate from the program.

In year 1, the majority of applicants did not propose implementation science topics. Therefore, in the subsequent application years, we adopted a new proposal format developed through collaboration between the Malawi HIV implementation Research Scientist Training (MHIRST) program, Sub-Saharan Africa Regional Partnership (SHARP) mental health implementation science, and NCD-BRITE. Specifically, the research proposal format requested Implementation Science elements using the following components: objective, public health importance, evidence-based intervention, context, barriers, implementation strategies, study design, outcomes, and public health impact. Additionally, the “Introduction to Implementation Science” short course preceded the grant application deadline so that applicants were well-versed in implementation science principles and the proposed grant format. These modifications resulted in stronger proposals aligned with the goals of the grant.

Many applicants are disqualified because they do not follow grant submission instructions. We therefore take this as opportunity to share learning moments, i.e., there is need to mentor candidates on grant application process especially as they interact with their potential mentors, applicants need to start compiling their applications well in advance and to have enough time to discuss with their potential mentors. This will allow them to be able to quality check the application for references, formatting, supporting documents the appropriateness of the forms used and any other guidelines that may be required. Proposal submissions internationally often have strict deadlines and guidelines and require properly referenced scientific argument and upcoming researchers must start to get used to such habits in order to make their research training more effective.

## Challenges

While our mentored initiative is able to support direct research costs, it cannot support investigator (mentees) salaries. The mentees must therefore have work-release by their main employer without affecting their earning capacity to allow for time for the research mentorship. Where the mentor and mentee work for the same institution, it is often easier to make arrangements for mentorship. However, such scenarios although they do occur are not specifically encouraged as mentor-mentee matching should be done based on mutual research interests rather than being from the same institution. An important component of the NCD BRITE program is to engage all partners working in the NCD area to ensure involvement of our mentees in funded activities where possible and engage mentors working in this area.

In terms of disease conditions selected for research mentorship, hypertension and diabetes are the popular topics. Two physicians are working on a proposal for heart failure and one non-physician researchers is working on asthma. There are no researchers in COPD, perhaps reflecting the paucity of mentors in this area.

## Conclusions

Our mentored research strategy has potential to build NCD research capacity and may be a launch pad for junior researchers as they become independent principal investigators. Identifying the right candidates who will be willing and able to continue in the NCDs’ research arena is critical. The NCD BRITE initiative provides a workable mentorship model for capacity building programs for NCD research practice in resource limited settings like Malawi. Our final project report will highlight this model and will report recommendations that may be necessary for a successful training program in the NCDs’ field in similar settings.

## Data Availability

Not applicable.

## Abbreviations

BRITE: Building Research Capacity, Implementation, and Translation Expertise
HRH: Human resources for health
NCD: Non-communicable diseases
QECH: Queen Elizabeth Central Hospital
